# Vinegar-baked *Radix Bupleuri* modulates the cell membrane constituents and inhibits the P-gp activity in rat hepatocytes

**DOI:** 10.1186/1472-6882-14-357

**Published:** 2014-09-25

**Authors:** Ruizhi Zhao, Lijuan Liu, Yinjie Wang, Zhicai Xiao

**Affiliations:** Second Affiliated Hospital, Guangzhou University of Chinese Medicine, Neihuan Xilu, Guangzhou Daxuecheng, Guangzhou 510006 China

**Keywords:** *Radix Bupleuri*, P-gp, Phosphosphingolipid, Phosphatidylserine, Phosphatidylcholine, Phosphatidyl ethanolamine, Vinegar, Membrane constituents, Cholesterol

## Abstract

**Background:**

Vinegar-baked *Radix Bupleuri* (VBRB) enhances the effects of other drugs on the liver by increasing drug distribution to the liver, but the mechanism of action remains unclear. The present study was designed to determine the effects of VBRB on the membrane permeability, constituents, and P-glycoprotein (P-gp) activity of hepatocyte BRL cells, in order to interpret the liver targeting enhancing effects of VBRB.

**Methods:**

The membrane permeability and P-gp expression were analyzed by flow cytometry. The membrane constituents were determined by an automatic biochemistry analyzer and thin-layer chromatography.

**Results:**

The results showed that, compared with the control, VBRB enhanced the membrane permeability by 41-67% (P < 0.05), which occurred in the absence of any cytotoxicity. VBRB had marginal effects on the cholesterol content, but significantly affected the total protein contents and the lipid constituents of the cell membrane in a dose- and time-dependent manner. VBRB inhibited P-gp expression in the cell membrane by 59-86% (P < 0.01).

**Conclusion:**

VBRB affects the constituents of BRL cells and increases its permeability, which may help explain its liver-targeting effects.

## Background

*Bupleurum Chinense DC* (Bei Chai Hu, *Radix Bupleuri)* has a wide spectrum of pharmacological effects [1–3], and is a frequently used herb in China for treating various diseases, such as fever, influenza, menstrual disorder, jaundice, bitter taste in the mouth as well as hypochondriac pain and hepatitis. Vinegar-baked *Radix Bupleuri* (VBRB) is the processed product of *Radix Bupleuri* with vinegar and is commonly used for the treatment of liver diseases such as jaundice and hepatitis [4]. However, the mechanisms of action of VBRB are not fully understood.

Drug efficacy is primarily dependent on its concentration at the site of action. In our previous studies, resveratrol, rhein and oxymatrine were selected as model drugs to investigate the effects of VBRB on drugs distribution. Our results showed that VBRB increased the liver distribution of resveratrol, rhein, and oxymatrine [5–7] in experimental rats or mice, indicating that VBRB may enhance liver uptake or decrease the efflux of these drugs.

Drug distribution is often determined by the interactions between drug and its microenvironment, including transporters and the constituents of cell membrane. Therefore, in the present study, the influence of VBRB on the permeability of the cell membrane of normal rat liver cell line BRL was investigated. Furthermore, the effects of VBRB on membrane components and P-glycoprotein (P-gp) expression were analyzed. These results are beneficial to the rational use of VBRB to improve its clinical efficacy. .

## Methods

### Chemicals

VBRB [*Radix* of *Bupleurum Chinense DC*, baked with 20% vinegar, or as cited as B Chinese in the Pharmacopeia of China (Bei Chai Hu)] was purchased from Kangmei Medical Company (Guangzhou, China), and was produced in Hubei province, China (batch number 20070120). The sample was authenticated and voucher specimens were deposited in the Department of Pharmacognosy and Phytochemistry, Guangzhou University of Chinese Medicine. Dextran labeled with fluorescein isothiocyanate (FD-500S), 3-(4, 5-Dimethylthiazol-2-yl)-2, 5-diphenyltetrazolium bromide (MTT), Dimethyl sulfoxide (DMSO), N-2-Hydroxyethylpiperazine -N—2- ethanesulfonic acid (HEPES), phenylmethylsulfonyl fluoride (PMSF), Tris, penicillin, and streptomycin were purchased from Sigma-Aldrich (St. Louis, MO, USA). Dulbecco’s modified Eagle’s medium (DMEM) in high glucose, 0.25% pancreatin, and new-born calf serum were purchased from Gibco Company (Grand Island, USA). Phosphate buffered saline (PBS) was purchased from Dingguo Biology and Technology Company (Beijing, China). Phosphosphingolipid (SM, 98%), phosphatidylserine (PS, 98%), phosphatidylcholine (PC, 98%), and phosphatidyl ethanolamine (PE, 98%) were obtained from LARODAN (Malmo, Sweden). Prefabricated plates were obtained from Merck (Darmstadt, Germany). CD 243 was purchased from Beckman Coulter (Fullerton, USA).

### Extraction of VBRB

The extraction of VBRB was carried out according to the method reported previously [5]. Briefly, 50 g of VBRB was soaked in 500 mL of water for 0.5 h, heated to boiling point, and kept boiling for 45 min, and then filtered. The herbs were extracted again with 400 mL of water using the above procedure. The pooled filtrates were condensed to 100 mL by rotary evaporation at 70°C, and then stored at −20°C until use. The percent was based on the dry weight of polysaccharides or saponin in the total weight of VBRB and was determined in our laboratory. There were about 4.5% of high molecular weight polysaccharides, 3.5% of low molecular weight polysaccharides, and 1% of sum-saponin and sapogenin in VBRB. The concentrations of saikosides were determined by HPLC-MS in our laboratory and the contents of Saikoside a, b, c, d in the extract were 44.45, 79.3, 10.6, and 43.3 mg, respectively.

### Cell line and culture

Normal rat hepatocyte cell line BRL was purchased from the Cell Research Center of Chinese Academy of Science (Shanghai, China). The cells were maintained with DMEM containing 10% new-born calf serum, 0.25% pancreatic enzyme (Gibco, Grand Island, USA), 100 ng/mL of penicillin, and 100 ng/mL of streptomycin at 37°C in a humidified atmosphere containing 5% CO2.

### Cell viability assay

BRL Cells were seeded onto 96-well plates at a density of 2 × 10^5^ cells/mL. After incubation for 24 h at 37°C and 5%CO_2_, 150 μL of VBRB solution at different concentrations (0.16, 0.8, 4.0, 20, and 100 mg/mL) was added and co-cultured for 24 h; and then 20 μl of MTT solution (5 mg/ml) was added into each well and incubated at 37°C for 4 h. Then the supernatant was removed and 150 μl of DMSO was added to solubilize the formazan products. Absorbance was measured at the wavelength of 570 nm. The control wells were designated as 100% viability. The blank values, indicating the absorbance of MTT and DMSO only, were subtracted from all the samples.

### Membrane permeability study

Viable BRL cells (2 × 10^5^/mL) were washed with PBS twice, suspended in Gibco RPMI 1640 (Gibco, Grand Island, USA) and incubated in 6-well plates. After a 24-h incubation, VBRB at various concentrations (0, 0.4, 2, and 10 mg/mL) was added and co-cultured. After 3 and 6 h of co-culture, cells were washed with PBS twice. Then, DMEM containing 10% new-born calf serum and 20 μl (5 mg/mL) FD-500S was added and cultured for 2 h, thereafter, 0.25% pancreatic enzyme was added, followed by trypsinization. The cell suspension was then centrifuged at 109 g for 5 min. Fluorescence staining of the positive cells (FD-500S taken up by cells) was quantified by flow cytometry (Elite Beckman coulter, Fullerton, USA) with emission and excitation wavelengths of 520 nm and 480 nm, respectively. 10,000 cells in each sample were counted and the percentage of positive cells was calculated.

### Determination of the constituents of the BRL membrane

Cell membrane extraction was carried out according to the methods reported previously [8]. In short, a cell suspension was prepared in a 75-cm^2^ flask, to which the VBRB extract at three different concentrations as aforementioned was added after cells were cultured for 24 h. After a 3- or 6-h culture with VBRB, the cells were washed twice with PBS and then suspended in 5 mL of 4°C HEPES buffer (1.2096 g/L) containing 0.1932 g/L MgCl_2_ and 0.0978 g/L PMSF. The cell suspension was then sonicated and centrifuged at 109 g for 10 min. The supernatant was further centrifuged at 12,000 g; the residue was resuspended in Tris–HCl (20 mM) buffer containing EDTA (1 mM) and PMSF (1 mM). The suspension-centrifugation procedure was then repeated an additional three times; the entire procedure was performed at 4°C. The final residue was used for subsequent analysis.

### Determination of total protein and cholesterol

The above mentioned residue was divided into two parts. Part I was dissolved in 1 mL of HEPES buffer for total protein analysis and Part II was dissolved in 1 mL of dehydrated alcohol for cholesterol determination. Both samples were centrifuged at 1760 g for 10 min, and the supernatant was collected and used for analysis by automatic biochemistry analyzer (PE Company, Waltham, USA) [9, 10].

### Determination of membrane lipids

The total lipids were the sum of typical lipids that were determined by the HPTLC scanning method [11], and then the sum of the four lipids was calculated accordingly. In brief, according to the method reported by Ismaili et al. [11], thin-layer chromatography was used for the determination of membrane lipids. Residue prepared above was dissolved in 1 mL of ethanol for analysis. The standard solutions of phosphosphingolipid (SM), phosphatidylcholine (PC), phosphatidyl ethanolamine (PE) and phosphatidylserine (PS) were prepared at concentrations 5, 10, 10 and 25 mg/mL, respectively, and sample solutions were spotted on a plate (Merck, Germany). The spotting volume was 5 μL for samples and 1 or 4 μL for standard solutions. Afterwards, the plate was developed in an automatic developing chamber (Camag, Switzerland) at 25°C with 75% humidity and a developing distance of 8 cm. The development solution was chloroform-methanol-acetic acid- water (10:2:1:0.25). After development, the plate was dried and placed in a chamber of iodine vapor for 5 min. Finally, the plate was covered with a blank plate and scanned at 380 nm with a Shimadzu (Japan) CS-9301 densitometric scanner, using the software provided with the instrument, and the peak areas were recorded. The peak areas obtained from the standard solution were plotted against the quality of the four components to produce separate working calibration plots for each compound. The amounts of each lipid in the samples were then calculated by use of the respective calibration plots.

### P-gp expression determination

After a 3- or 6-h culture with various concentrations of VBRB as noted above, the cells were washed twice with PBS and then suspended in 500 μL PBS. Afterwards, 20 μL of fluorescein for labeling P-gp (CD 243) was added. After a 15-min incubation, the free CD 243 was washed with 1 mL PBS and the fluorescein stain ratio determined by a flow cytometry (Elite Beckman coulter, USA) using emission and excitation wavelengths of 520 nm and 480 nm, respectively.

### Statistical analysis

The data are presented as the mean and standard deviation (*X* ± S). All statistical analyses were performed using SPSS 11.0 for Windows. The differences among various treatments were analyzed by ANOVA using log *X* and Q test for statistical analyses. P < 0.05 was regarded as statistically significant.

## Results

### VBRB increases cell membrane permeability

**Figure 1:**
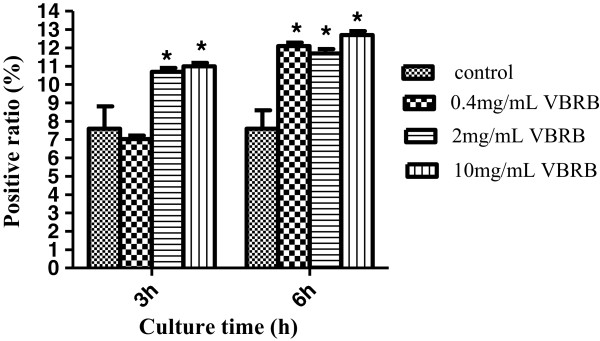
**Effect of VBRB on the permeability of BRL cells.**

As shown in Figure 1, almost all of the VBRB groups showed significantly increased membrane permeability in BRL cells, compared with control. The increased permeability values were between 41%-67%, except for the lowest concentration group after a 3-h culture. As the culture time prolonged, the permeability increased significantly (P < 0.05); the permeability at 24 h was 1.5 times higher than that of the control group (data not shown). A concentration-dependent increase in permeability was also observed after a 3-h culture (P < 0.05); however, no significant difference was found among the different VBRB groups after a 6-h culture.

### Cell viability

**Figure 2:**
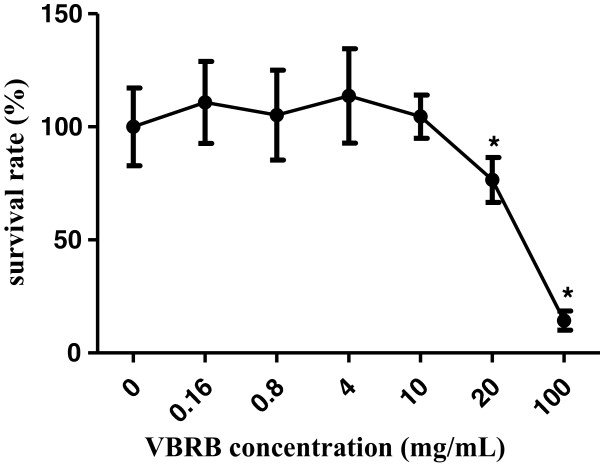
**Effect of VBRB on viability of BRL cells.**

Considering that an increase in cell membrane permeability may be related to drug toxicity, several concentrations of VBRB were used in our cell viability study in order to determine if the increase in membrane permeability is due to the toxicity of VBRB. As shown in Figure 2, VBRB inhibited cell proliferation significantly when the VBRB concentration was higher than 20 mg/mL; however, when VBRB concentration was lower than 10 mg/mL, VBRB had a marginal effect on cell viability, indicating that increased permeability was not induced by a broken cell membrane.

### VBRB has marginal effects on cholesterol contents

**Figure 3:**
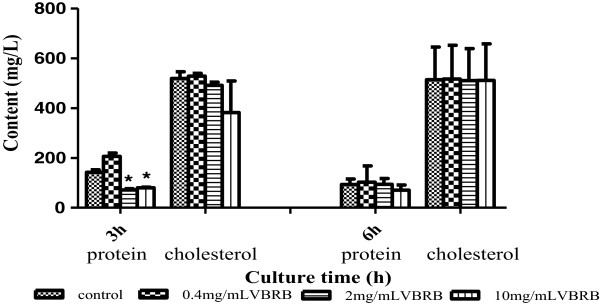
**Effect of VBRB on the contents of total protein and cholesterol.**

Changes in cell membrane constituents may affect permeability. Therefore, the effects of VBRB on membrane constituents were studied. As shown in Figure 3, the total cholesterol content remained stable in the control cells, and VBRB had marginal effects on total cholesterol content.

### VBRB has effects on total protein contents in a time-dependent manner

Unlike total cholesterol, VBRB affected the total protein contents in a time-dependent manner. After a 3-h culture, the medium and high concentrations of VBRB, but not the low concentration, decreased total protein significantly (P < 0.05). The decreased total protein extent was 50% and 44% for the medium and high VBRB doses, respectively. After a 6-h culture, all three concentrations of VBRB showed marginal effects on protein contents (Figure 3).

### VBRB has significant effects on membrane lipids

The analytical method for lipids was validated by determining the specificity, linearity, accuracy, precision, and stability. All four lipids (SM, PS, PC and PE) were separated well in the given analytical conditions and a good linearity was shown for each of the lipids. The calibration curves for SM, PS, PC and PE were Y = 216.0X-49.19 (R^2^ = 0.993), Y = 223.4X + 19.38 (R^2^ = 0.995), Y = 127.6X + 47.68 (R^2^ = 0.991), and Y = 88.49X + 67.97 (R^2^ = 0.995), respectively. The detection limits for SM, PS, PC and PE were 2.5, 5, 5, 12.5 μg, respectively, and the linear range was 2.5-30, 5–60, 5–60, 12.5-150 μg, respectively. The spots were stable between 0.5-3 h with the SD being below 4.5% and the RSDs of precision and accuracy below 3.4% and 3.7%, respectively.

**Figure 4:**
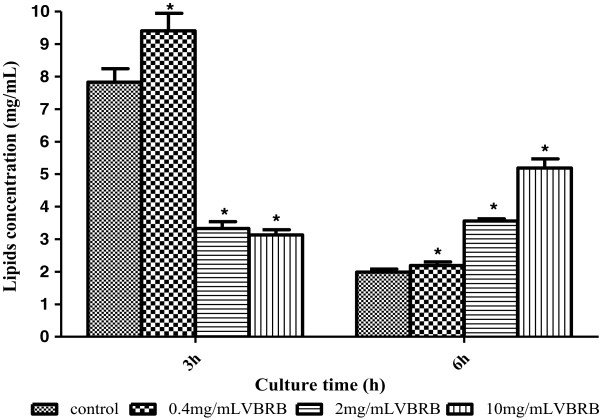
**Effect of VBRB on the total lipids.**

The sum of the four lipids (SM, PS, PC and PE) was calculated as the total lipids content. The effects of VBRB on the total lipids were dependent on culture time and VBRB concentration (Figure 4). At low concentration, VBRB increased the total lipids at both 3- and 6-h culture times; at the medium and high concentration, VBRB decreased the total lipids content at the 3-h culture time point, but increased the total lipids content at 6-h culture time significantly (P < 0.05).

**Figure 5:**
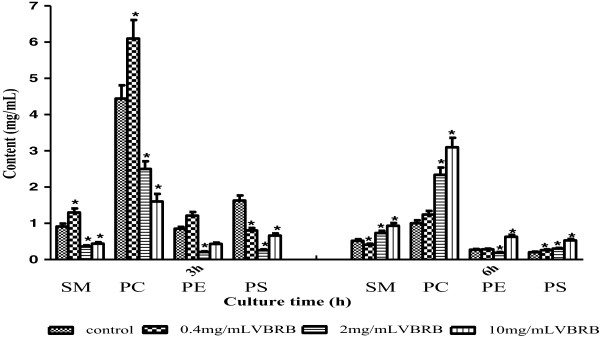
**Effect of VBRB on specific lipid contents.**

We further examined four major types of lipids in BRL cells after VBRB treatment and found that the specific lipid contents were affected in a type-of-lipids-, culture time-, and VBRB concentration-dependent manner (Figure 5). At 3 h after culture, the medium and high concentrations of VBRB decreased the contents of all the four lipids significantly (P < 0.05). In contrast, at low concentration, VBRB decreased the content of PS, but increased the contents of SM and PC significantly (P < 0.05). However, after a 6-h culture the low concentration of VBRB decreased the content of SM but increased the content of PS significantly (P < 0.05). The medium and high concentrations of VBRB increased the contents of all the four lipids significantly, except for the medium concentration of VBRB, which decreased the PE content significantly (P < 0.05).

### VBRB inhibits P-gp expression

**Figure 6:**
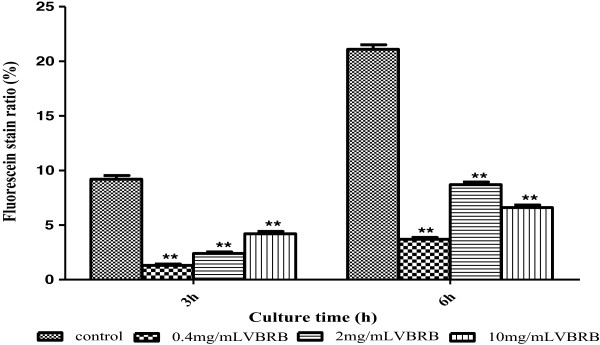
**Effect of VBRB on P-gp expression in BRL cells.**

P-gp is a transporter in the cell membrane and is responsible for the efflux of substrates of the Pgp, which is a key factor affecting the retention of many drugs in the cell. Therefore, we studied the effects of VBRB on P-gp expression. As shown in Figure 6, VBRB inhibited P-gp expression significantly at all the time points (P < 0.01); and the low concentration of VBRB had stronger effects than high concentrations. This result was in accordance with a previous study by Zhu et al., which reported that *Radix Bupleuri* increases the cell concentration of vincristine significantly, indicating that *Radix Bupleuri* inhibited the activity of P-gp in human liver cancer cell line BEL-7402 [12].

## Discussion

The action of drugs is often determined by the drug’s concentration at target sites such as organs and cells; and drug concentration in cells is a balance of uptake and efflux. Drugs uptake or efflux by cells are determined by many factors, such as the drug’s molecular size, polarity, charge, and, more importantly, its affinity to the cell membrane [13]. Usually, small molecules with good affinity to the cell membrane can be taken up or efflux out by diffusion; however, only very few molecules are transported through cells by this way. Most molecules utilize a transporter-mediated mechanism. The activity of transporters is affected by many factors, such as the amount and stereo-chemical structure of the proteins, which may be further affected by the lipids and cholesterol around it [14, 15]. The first sign reflecting the drug-induced changes in membrane microenvironment may be the changes in cell permeability. Therefore, in the present study, we first determined the effects of VBRB on cell permeability and found that VBRB enhanced the permeability of BRL cells significantly.

There are two types of hypotheses for the possible mechanism of increased permeability; one is aqueous pore formation and the other is a carrier-related mechanism [16]. In the former case, low molecular weight substances can get into cells easily and lead to membrane disruption and cell death. In the latter case, although cell membrane permeability is increased, cells are alive. In order to rule out that the membrane permeability is induced by drug toxicity, the effects of VBRB on the cell viability of BRL cells were determined in a broad range of VBRB concentrations. The results indicated that, at the concentrations used in the permeability study, VBRB had no or marginal effects on cell viability. Therefore, the increased permeability caused by VBRB was not due to aqueous pore formation and was most likely due to a carrier-dependent mechanism. Carriers are usually proteins; their activity is affected by protein content and its microenvironment, such as the contents of lipids and cholesterol [14, 15]. Therefore, the effects of VBRB on membrane constituents were further studied in the subsequent experiments.

Cholesterol is the skeleton of the cell membrane; decreasing the cholesterol content usually induces cell death [17]. Our results indicate that VBRB had a marginal influence on the content of cholesterol, indicating that VBRB at the test doses in this study does not affect the major cell membrane structure, which is helpful to maintain cell integrity.

Proteins are the major components of channels and transporters and their contents are an index of transporter activity. Our results indicate that VBRB decreased total protein content after a 3-h culture and had a marginal effect after a 6-h culture, implying that the effects of VBRB on protein content may be transient. Since total protein levels contain all kinds of membrane proteins, including two contrary direction transporters, uptake and efflux transporters, total protein levels may not be a good indicator for transporter studies such as the present study. The uptake transporters include organic anion transporting polypeptides, organic anion transporters, and organic cation transporters; the efflux pumps consist of P-gp, multidrug resistance-associated protein family, bile salt export pump, breast cancer resistance protein, and others. Therefore, further study of VBRB effects on specific protein content is essential.

Total lipid content reflects the polarity of the cell and is also a key element determining drug-membrane affinity and drug distribution. When total lipid content is increased, the polarity of the cell membrane is decreased; low polarity drugs such as resveratrol and rhein may then get into liver cells easily. When lipid content is decreased, the membrane polarity is increased and drugs with higher polarity may have a better affinity.

Besides polarity, the membrane lipids specifically or non-specifically interact with membrane proteins, affecting both the partitioning and folding of the protein and its function [15]. Some lipids may be incorporated into transporters and are necessary for the activity of transporters [18, 19]. Furthermore, all lipids are substrates of ATP-dependent ATP-binding cassette (ABC) translocators [20]. ABC transporters regulate the efflux and redistribution of lipids and, in turn, lipid composition can control ABC activity. This is proven by many studies [21–24]. Since there are many kinds of transporters in hepatocytes and the total drug uptake is the combined effect of all related factors, the effects of single specific lipids on the activity of given transporters needs to be further investigated.

A decrease in efflux activity is one of the major reasons for increased permeability. The results of this study show that VBRB inhibited the activity of P-gp significantly, indicating a prolonged retention time of drugs in the liver, which is in agreement with our previous results that VBRB decreases the clearance of the test compounds from the liver [5–7].

In traditional Chinese medicine, site-directed drug effects can be achieved by co-administration of therapeutic drugs with organ-targeting agents, leading to either synergistic or attenuation effects [4]. This phenomenon is described as meridian guide theory. VBRB is one of the meridian guide drugs for liver targeting, which can enhance the effects of other drugs on the liver [7]. Our previous study shows that this liver enhancing effect of VBRB is due to its effect on distribution of other drugs in the liver [5–7]. The present study first revealed that VBRB affected the microenvironment of the cell, which may be responsible for its site-directed effects.

## Conclusion

In the present study, we demonstrated that VBRB affected the permeability of cultured BRL cells in a time-dependent manner, which was not due to a broken membrane. Moreover, the effects of VBRB on membrane constituents were dependent on VBRB dose, action time and membrane constituent type. VBRB affected the contents of lipids and protein, but not cholesterol. VBRB inhibited P-gp expression significantly. In summary, affecting lipid contents and inhibiting P-gp expression are possible mechanisms for the enhanced liver targeting effects of VBRB, and close attention to drug-drug interactions should be paid when co-administering drugs with VBRB in the clinic.

## Abbreviations

VBRB: Vinegar-baked *Radix Bupleuri*
P-gp: P-glycoprotein
SM: Phosphosphingolipid
PC: Phosphatidylcholine
PE: Phosphatidyl ethanolamine
PS: Phosphatidylserine.
